# Surgery on admission and following day reduces hip fracture complications: a Japanese DPC study

**DOI:** 10.1007/s00774-024-01534-2

**Published:** 2024-07-11

**Authors:** Yu Mori, Kunio Tarasawa, Hidetatsu Tanaka, Naoko Mori, Kiyohide Fushimi, Kenji Fujimori, Toshimi Aizawa

**Affiliations:** 1 Department of Orthopaedic Surgery, Tohoku University Graduate School of Medicine, 1-1 Seiryo-machi, Aoba-ku, Sendai, Miyagi 980-8574 Japan; 2 Department of Health Administration and Policy, Tohoku University Graduate School of Medicine, 2-1 Seiryo-machi, Aoba-ku, Sendai, Miyagi 980-8574 Japan; 3 Department of Radiology, Akita University Graduate School of Medicine, 1-1-1 Hondo, Akita, 010-8543 Japan; 4 Department of Health Policy and Informatics, Tokyo Medical and Dental University Graduate School of Medicine and Dental Sciences, 1-5-45 Yushima, Bunkyo-ku, Tokyo, 113-8519 Japan

**Keywords:** Hip fracture, Mortality, Pneumonia, Pulmonary embolism, Surgery

## Abstract

**Introduction:**

The efficacy of early surgery in preventing complications among Japanese elderly patients with hip fractures requires further investigation. This study aims to use a comprehensive Japanese hip fracture case database to determine whether surgery within the day of admission and the following day reduces the incidence of complications and mortality during hospitalization in elderly hip fracture patients.

**Materials and methods:**

We retrospectively analyzed the Japanese National Administrative DPC (Diagnosis Procedure Combination) database from April 2016 to March 2022. Approximately 1100 DPC-affiliated hospitals consistently provided medical records with consent for research. The study investigated the association between postoperative pneumonia, deep vein thrombosis, pulmonary embolism, and mortality during hospitalization after propensity score matching, focusing on surgeries conducted on the day of admission and the following day.

**Results:**

After one-to-one propensity score matching for age, gender, and comorbidity, we identified 146,441 pairs of patients who underwent surgery either within the day of admission and the following day or after the third day of admission. Surgery on the third day or later was independently associated with increased risks of pneumonia, deep vein thrombosis, pulmonary embolism, and mortality during hospitalization with risk ratios of 1.367 (95% CI 1.307–1.426), 1.328 (95% CI 1.169–1.508), 1.338 (95% CI 1.289–1.388), and 1.167 (95% CI 1.103–1.234), respectively.

**Conclusion:**

A comprehensive study of elderly Japanese patients with hip fractures in the DPC database showed that surgery on admission and the following day is crucial for preventing complications like pneumonia, deep vein thrombosis, pulmonary embolism, and mortality during hospitalization.

## Introduction

Hip fracture is a common orthopedic injury in the elderly and is associated with high patient morbidity and mortality [1, 2]. As the elderly population continues to grow in the US, the incidence of hip fracture has doubled from 250,000 in 1990 to a projected 500,000 in 2040 [3]. In Japan, with an aging population, there are 13 million people with osteoporosis [4], and patients with hip fractures are estimated to be 250,000 [5]. Thus, hip fractures due to osteoporosis are a global problem, regardless of country or region. More than 90% of these fractures occur in patients aged 65 years and older. In patients with femoral neck fractures, pre-injury complications increase over time as well. A 30 day mortality rate of 4.0–5.4% [6] and an estimated 1 year mortality rate of 25% have been reported for femoral neck fractures [7].

There is a vast amount of literature reporting on timely hip fracture surgery concerning morbidity and mortality [8–11]. US and Canadian guidelines strongly recommend hip fractures within 48 h of surgery. This is because surgical delay beyond 48 h is associated with worse outcomes [8, 10, 11]; a meta-analysis and meta-regression of 35 studies including over 190,000 patients found that prevention of surgical delay beyond 48 h was associated with significant life extension and reduction in decubitus ulcers [9]. Based on these findings in Japan, additional reimbursement is now provided for hip fracture surgery within 48 h of injury [5]. On the other hand, no large-scale studies have reported whether surgery for hip fractures within 48 h reduces complications such as pneumonia, deep vein thrombosis, pulmonary embolism, and mortality during hospitalization in Japanese patients, and the benefit of early surgery remains unresolved. Although the risk of deep vein thrombosis and pulmonary embolization in blacks following trauma has been reported to be higher than in whites [12], no large-scale studies comparing Asians and whites have been reported, and research results are needed to support the efficacy of early surgery within 48 h in preventing subsequent events in Japanese patients.

Studies using the Diagnosis Procedure Combination (DPC) database in Japanese hip fracture cases have previously reported the impact of dementia [13] and the results of a study on the length of hospital stay [14]. On the other hand, the impact of early surgery has not yet been studied. Therefore, this study aims to determine, using a large database of Japanese hip fractures, whether surgery performed on the day of admission and the following day for hip fracture is associated with the occurrence of pneumonia, deep vein thrombosis, pulmonary embolism, and mortality during hospitalization sequelae in geriatric hip fracture patients.

## Materials and methods

### Study design

This retrospective study was conducted in accordance with the ethical standards of the Declaration of Helsinki and approved by the Tokyo Medical and Dental University (approval number: M2000-788). The Japanese National Administrative DPC reimbursement system database was retrospectively reviewed [15]. The study covered from April 2016 to March 2022. Throughout this period, approximately 1100 hospitals eligible under the DPC system consistently submitted their medical records and consented to their use for research purposes. Patients treated with hip fractures at these 1100 hospitals across Japan were enrolled in subsequent analyses. The data reflect the actual clinical practice in the country. Proximal femur fractures in the elderly were the focus of clinical research. Elderly persons were defined as those aged 65 years and over. Hip fractures were selected by the international statistical classification of disease-10 disease names. Three fractures of the proximal femur were included: fracture of the femoral neck (S7200), fracture of the femoral trochanteric (S7210), and fracture of the femoral subtrochanteric (S7220). For the hip fracture cohorts, patients were selected from a registry that included the following three categories: [1] principal diagnosis [2], principal reason for admission, and [3] disease requiring the most medical resources. To obtain a reliable hip fracture cohort, patients with postoperative proximal femur or localized fractures, e.g., only greater trochanter fractures, were excluded.

### Propensity score matching

We performed a one-to-one propensity score (PS) matching between surgical cases on the day of admission and the following day and those on the third day of admission or later. Covariates used for confounding adjustment included age, gender, and comorbidities such as hypertension, dementia, ischemic heart disease, cerebrovascular disease, chronic renal dysfunction, chronic lung disease, and diabetes. C-statistics were calculated to assess the discriminatory power of the model. PS estimates were used to perform nearest-neighbor matching without replacement, with the PS estimates being used as the calipers; the caliper was set to 0.2 times the standard deviation of the PS estimate. This resulted in matched pairs and established PS-matched control and treatment groups.

### Statistical analyses

All data are expressed as mean ± standard deviation. Significant differences between the two groups were examined using the Student’s t-test and χ^2^ test for each clinical parameter for the surgery on the day of admission and the following day and on or after the third-day group. The χ^2^ test was used to examine the association between delayed surgery and the occurrence of pneumonia, deep vein thrombosis, secondary pulmonary embolism, and mortality during hospitalization. The association between age, gender, comorbidity, general anesthesia, and early surgery and their impact on pneumonia, deep vein thrombosis, pulmonary embolism, and mortality during hospitalization were examined using multivariate logistic regression analysis. All statistical tests were two-tailed, with p-values < 0.001 considered statistically significant. All analyses were performed using JMP, version 17 (SAS, Cary, NC, USA).

## Results

**Fig. 1:**
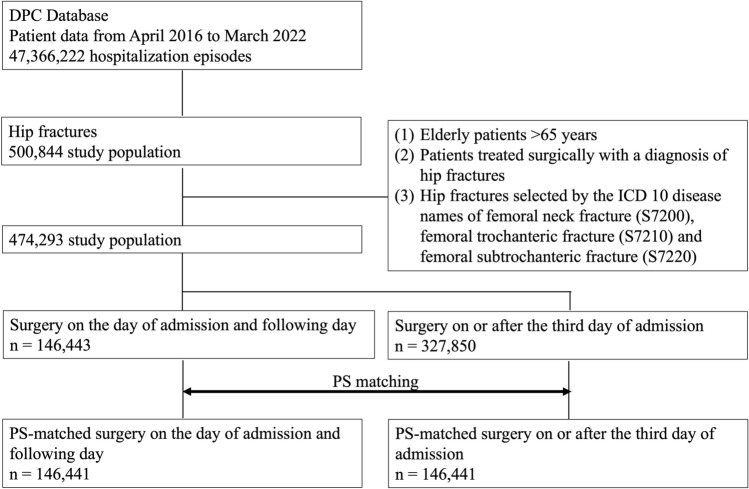
Flow diagram of patient selection for elderly hip fracture and PS matching. This diagram shows how eligible patients were extracted from the DPC database and the actual PS matching between patients of surgery on the day of admission and the following day and surgery on or after the third day of admission

The patient selection process is shown in Fig. 1 : a total of 474,293 patients meeting inclusion and exclusion criteria were selected from patient data from April 2016 to March 2022. Of these patients, 146,443 had surgery within the day of admission and the following day, and 327,850 had surgery on the third day of admission or later. After PS matching by age, gender, and comorbidity, there were 146,441 cases in each group, respectively. The C statistic was 0.7261. The mean wait for surgery for elderly hip fracture patients in this study was 2.7 ± 3.7 days, with a median of 1.5 days, first quartile of 1 day, and third quartile of 4 days. The mean wait for surgery for the group that had surgery on the day of admission and the following day was 0.7 ± 0.5 days, with a median of 1 day, the first quartile of 0 days, and the third quartile of 1 day. The mean and median wait for surgery for the group that underwent surgery on or after the third day after admission was 4.7 ± 4.3 days and 4 days, respectively; the first quartile was 3 days, and the third quartile was 6 days. The characteristics of surgical cases on the day of admission and the following day and cases on or after the third day are shown in Table 1. There were no significant differences between the two groups in the parameters of the adjustment factors for propensity score matching. Standardized mean differences were less than 0.1 for all parameters. The body mass index tended to be higher in the early surgery group. The group undergoing early surgery exhibited a higher incidence of femoral trochanteric fractures, whereas the group with delayed surgery presented more femoral neck fractures. In the group where surgery was performed on the day of admission or the next day, 80.1% of cases started rehabilitation within the third day of admission. Even in the group that underwent surgery on or after the third day of hospitalization, more than 65% of cases received rehabilitation within the third day of hospitalization, although it was impossible to determine whether the rehabilitation was pre- or post-operative.

**Table 1:** Characteristics of patients after propensity score matching

After propensity score matching				
	Surgery on the day of admission and following day	Surgery on or after the third day of admission	SMD	*p*-value
n	146,441	146,441		
Age	84.8 ± 7.7	84.8 ± 7.6	0.0008	0.85
Gender				
Men	30,062 (20.5%)	30,122 (20.6%)	0.001	0.78
Women	116,379 (79.5%)	116,319 (78.4%)		
Comorbidities				
Hypertension	53,942 (36.8%)	53,856 (36.8%)	0.0012	0.75
Dementia	32,046 (21.9%)	31,969 (21.8%)	0.0012	0.73
Diabetes	24,441 (16.7%)	24,431 (16.7%)	0.0002	0.96
Cerebrovascular disease	13,322 (9.1%)	13,287 (9.1%)	0.0008	0.82
Ischemic heart disease	10,356 (7.1%)	10,310 (7.0%)	0.0012	0.74
Chronic renal dysfunction	6534 (4.5%)	6557 (4.5%)	0.0008	0.84
Chronic lung disease	1914 (1.3%)	1872 (1.3%)	0.0026	0.49

			χ^2^ statics	*p*-value
Body mass index	20.52 ± 5.5	20.47 ± 4.1		0.008
Fracture types				
Femoral neck	62,086 (42.4%)	77,695 (53.1%)	3482	< 0.0001
Trochanteric	81,216 (55.5%)	65,484 (44.7%)		
Subtrochanteric	3139 (2.1%)	3262 (2.2%)		
Rehabilitation				
Rehabilitation within the third day of admission	117,363 (80.1%)	50,591 (65.4%)	64,911	< 0.0001
Rehabilitation on or after the fourth day of admission	29,078 (19.9%)	95,850 (34.6%)		

Data are shown as mean ± standard deviation

*SMD* standard mean difference

*p*-values of < 0.001 are considered significant by Student’s t-test and the χ^2^ test

Table 2 displays the usage of anticoagulants and medications for osteoporosis. The administration of edoxaban tosilate hydrate, fondaparinux sodium, and enoxaparin sodium, which are anticoagulants used to prevent deep vein thrombosis and pulmonary embolism following a hip fracture, was notably prevalent in the group that underwent surgery on or after the third day of admission. Additionally, other anticoagulants prescribed for treating comorbidities were more frequently used in the group that underwent surgery on or after the third day of admission. Osteoporosis was often treated with bisphosphonates and an active form of vitamin D. However, it was clear that the osteoporosis treatment rate was low even in the cases with hip fractures.

**Table 2:** Anticoagulation and osteoporosis treatment

	Surgery on the day of admission and following day (%)	Surgery on or after the third day of admission (%)	χ^2^ statics	*p*-value
Anticoagulants				
All anticoagulants	64,783 (44.2)	73,717 (50.3)	1094	< 0.0001
Edoxaban tosilate hydrate	34,903 (23.8)	40,095 (27.4)	483.5	< 0.0001
Fondaparinux sodium	1989 (1.4)	2055 (1.4)	1.1	0.3
Enoxaparin sodium	5238 (3.6)	5874 (4.0)	37.9	< 0.0001
Aspirin	15,623 (10.7)	17,127 (11.7)	77.8	< 0.0001
Warfarin potassium	3978 (2.7)	6241 (4.3)	523.4	< 0.0001
Other anticoagulants	9892 (6.8)	12,115 (8.3)	243.2	< 0.0001
Osteoporosis treatment				
Daily bisphosphonates	341 (0.2)	300 (0.2)	2.6	0.1
Weekly bisphosphonates	11,347 (7.8)	10,333 (7.1)	51.2	< 0.0001
Monthly bisphosphonates (oral)	4397 (3.0)	3461 (2.4)	114.8	< 0.0001
Monthly bisphosphonates (iv)	1151 (0.8)	1030 (0.7)	6.8	0.009
Yearly bisphosphonates (iv)	207 (0.1)	258 (0.2)	5.6	0.018
Daily teriparatide	1372 (1.0)	1663 (1.1)	28.2	< 0.0001
Weekly teriparatide	702 (0.5)	1027 (0.7)	71.3	< 0.0001
Biweekly teriparatide	261 (0.2)	221 (0.2)	4.3	0.04
Denosumab	761 (0.5)	459 (0.3)	75.8	< 0.0001
Eldecalcitol	14,865 (10.2)	13,029 (8.9)	133.7	< 0.0001
Alfacalcidol	16,037 (10.9)	15,058 (10.3)	34.5	< 0.0001
SERM	2263 (1.6)	2000 (1.4)	16.5	< 0.0001

*SERM* selective estrogen receptor modulator

*p*-values of < 0.001 are considered significant by the χ^2^ test; iv means intravenous injection

The association between surgery on or after the third day of admission and the development of pneumonia, deep vein thrombosis, pulmonary embolism, and mortality during hospitalization is shown in Table 3. For hip fractures, it was revealed that the risk of developing pneumonia, deep vein thrombosis, pulmonary embolism, and mortality during hospitalization increased to 1.354 (95% Confidence Interval [CI] 1.297–1.413), 1.331 (95% CI 1.173–1.510), 1.327 (95% CI 1.279–1.377) and 1.156 (95% CI 1.094–1.222) respectively when surgery was performed on or after the third day of admission.

**Table 3:** Association between occurrence of pneumonia, deep vein thrombosis, pulmonary embolism, and mortality during hospitalization and surgy on or after the third day of admission

Complication	Total (n)	Risk ratio (95% CI)	χ^2^ statics	*p*-value
Pneumonia	8711	1.354 (1.297–1.413)	191.8	< 0.0001
DVT	983	1.331 (1.173–1.510)	19.8	< 0.0001
PE	11,943	1.327 (1.279–1.377)	227.2	< 0.0001
Mortality during hospitalization	5082	1.156 (1.094–1.222)	26.3	< 0.0001

*PE* pulmonary embolism, *CI* confidence interval, *DVT* deep vein thrombosis

*p-values* of < 0.001 are considered significant by the χ^2^ test

The results of a multivariate logistic analysis of the association between pneumonia and age, gender, surgery on or after the third day of admission, and comorbidities are presented in Table 4. For pneumonia in hip fracture patients, male gender: 3.074 (95% CI 2.937–3.219); surgery on or after the third day of admission: 1.367 (95% CI 1.307–1.426); chronic lung disease: 2.244 (95% CI 1.995–2.523); dementia: 1.625 (95% CI 1.551–1703); and cerebrovascular disease: 1.374 (95% CI 1.289–1.465) were found to be the significant risks. In contrast, the general anesthesia was not the apparent risk of the pneumonia.

**Table 4:** Multivariate logistic analysis of risk factors for pneumonia**.**

Variable	Risk ratio (95% CI)	χ^2^ statics	*p*-value
Age	1.047 (1.044–1.050)	872.4	< 0.0001
Gender (Male)	3.074 (2.937–3.219)	2111	< 0.0001
Surgery on or after 3rd day	1.367 (1.307–1.426)	197.3	< 0.0001
General anesthesia	0.973 (0.932–1.018)	1.4	0.24
Hypertension	1.038 (0.993–1.086)	2.7	0.1
Diabetes	1.006 (0.949–1.067)	0.04	0.83
Cerebrovascular disease	1.374 (1.289–1.465)	88.3	< 0.0001
Chronic renal dysfunction	1.015 (0.920–1.119)	0.09	0.77
Ischemic heart disease	0.999 (0.920–1.119)	0.001	0.98
Dementia	1.625 (1.551–1.703)	390.8	< 0.0001
Chronic lung disease	2.244 (1.995–2.523)	151.1	< 0.0001

*CI* confidence interval

*p-values* of < 0.001 are considered significant by the χ^2^ test

The results of the multivariate logistic analysis for assessing risk factors for deep vein thrombosis are shown in Table 5. For deep vein thrombosis in hip fracture patients, surgery on or after the third day of admission: 1.328 (95% CI 1.169–1.508) and hypertension: 1.369 (95% CI 1.205–1.556) were shown to be the significant risks.

**Table 5:** Multivariate logistic analysis of risk factors for deep vein thrombosis

Variable	Risk ratio (95% CI)	χ^2^ statics	*p*-value
Age	1.005 (0.996–1.013)	1.3	0.26
Gender (Female)	1.049 (0.891–1.235)	0.3	0.56
Surgery on or after 3rd day	1.328 (1.169–1.508)	19.2	< 0.0001
General anesthesia	1.023 (0.899–1.164)	0.1	0.73
Hypertension	1.369 (1.205–1.556)	22.8	< 0.0001
Diabetes	1.087 (0.922–1.281)	1.0	0.32
Cerebrovascular disease	0.952 (0.765–1.186)	0.2	0.66
Chronic renal dysfunction	0.563 (0.381–0.833)	9.9	0.0016
Ischemic heart disease	1.394 (1.128–1.722)	8.7	0.003
Dementia	1.052 (0.905–1.222)	0.43	0.51
Chronic lung disease	1.290 (0.784–2.123)	0.9	0.33

*CI* confidence interval

*p-values* of < 0.001 are considered significant by the χ^2^ test

The outcomes from the multivariate logistic regression analysis aimed at identifying risk factors for pulmonary embolism are presented in Table 6. In the context of hip fracture patients, undergoing surgery on or after the third day of admission was associated with a risk ratio of 1.338 (95% CI 1.289–1.388), and hypertension was associated with a risk ratio of 1.467 (95% CI 1.414–1.524), both identified as significant risk factors. Female gender is associated with a higher risk of pulmonary embolism with a risk ratio of 1.340 (95% CI 1.273–1.410). Additionally, dementia was also associated with a higher risk of pulmonary embolism with a risk ratio of 1.152 (95% CI 1.103–1.203).

**Table 6:** Multivariate logistic analysis of risk factors for pulmonary embolism

Variable	Risk ratio (95% CI)	χ^2^ statics	*p*-value
Age	0.993 (0.990–0.995)	32.1	< 0.0001
Gender (Female)	1.340 (1.273–1.410)	133.7	< 0.0001
Surgery on or after 3rd day	1.338 (1.289–1.388)	236.4	< 0.0001
General anesthesia	0.941 (0.906–0.977)	10.1	0.0015
Hypertension	1.467 (1.414–1.524)	395.4	< 0.0001
Diabetes	0.958 (0.911–1.007)	2.9	0.09
Cerebrovascular disease	0.813 (0.759–0.871)	36.7	< 0.0001
Chronic renal dysfunction	0.838 (0.760–0.924)	13.3	0.0003
Ischemic heart disease	0.959 (0.892–1.032)	1.2	0.3
Dementia	1.152 (1.103–1.203)	39.9	< 0.0001
Chronic lung disease	0.862 (0.719–1.034)	2.7	0.1

*CI* confidence interval

*p-values* of < 0.001 are considered significant by the χ^2^ test

The outcomes from the multivariate logistic regression analysis aimed at identifying risk factors for mortality during hospitalization are presented in Table 7. For hip fracture patients, having surgery on or after the third day of admission was associated with a risk ratio of 1.167 (95% CI 1.103–1.234), and diabetes with a risk ratio of 1.232 (95% CI 1.145–1.326), chronic renal dysfunction with a risk ratio of 2.037 (95% CI 1.848–2.247), dementia with a risk ratio of 1.130 (95% CI 1.059–1.206), and chronic lung disease with a risk ratio of 2.211 (95% CI 1.900–2.572) were also distinctly associated, all identified as significant risk factors. Male gender is associated with a higher risk of mortality with a risk ratio of 2.715 (95% CI 2.557–2.882).

**Table 7:** Multivariate logistic analysis of risk factors for mortality during hospitalization

Variable	Risk ratio (95% CI)	χ^2^ statics	*p*-value
Age	1.067 (1.062–1.071)	1006.7	< 0.0001
Gender (Male)	2.715 (2.557–2.882)	976.2	< 0.0001
Surgery on or after 3rd day	1.167 (1.103–1.234)	29.0	< 0.0001
General anesthesia	0.932 (0.880–0.987)	5.9	0.015
Hypertension	0.758 (0.713–0.805)	82.5	< 0.0001
Diabetes	1.232 (1.145–1.326)	30.1	< 0.0001
Cerebrovascular disease	1.003 (0.912–1.103)	0.005	0.95
Chronic renal dysfunction	2.037 (1.848–2.247)	172.0	< 0.0001
Ischemic heart disease	1.040 (0.939–1.153)	0.56	0.45
Dementia	1.130 (1.059–1.206)	13.4	0.0003
Chronic lung disease	2.211 (1.900–2.572)	86.5	< 0.0001

*CI* confidence interval

*p-values* of < 0.001 are considered significant by the χ^2^ test

## Discussion

The study aimed to determine, using a large database of hip fractures in elderly Japanese patients, whether surgery performed on the day of admission and the following day was associated with the occurrence of sequelae of pneumonia, deep vein thrombosis, pulmonary embolism, and mortality during hospitalization in elderly hip fracture patients. The study results revealed that surgery performed on or after the third day of hospitalization leads to a 1.367-fold increased risk of pneumonia, 1.328-fold increased risk of deep vein thrombosis, 1.338-fold increased risk of pulmonary embolism, and 1.167-fold increased risk of mortality during hospitalization in a multivariate analysis in a population-adjusted for confounding factors, indicating an important role of surgery on the day of admission and the following day in patient outcomes in a Japanese elderly population.

In Japan, over 13 million individuals are affected by osteoporosis, and the number of hip fracture incidents is anticipated to rise from 240,000 in 2020 to 320,000 by 2040, the highest projection among Asian countries [16]. The economic impact of fragility fractures on the nation exceeds USD 10 billion, a figure expected to escalate with the aging population [5]. A study by the Japanese Orthopaedic Association highlights an average waiting period of four days for hip surgery across Japan, suggesting considerable and avoidable surgical delays [17]. Moreover, a recent analysis utilizing medical insurance data revealed that only 32.7% of patients received osteoporosis treatment post-hip fracture, underscoring a gap in secondary fracture prevention for patients at very high risk [18]. Implementing a Fracture Liaison Service, a multidisciplinary approach designed to identify and manage patients with fragility fractures has demonstrated effectiveness in significantly reducing rates of subsequent fractures and associated costs [19].

In a population of elderly patients with hip fractures, early rehabilitation leads to weaning. It effectively prevents pneumonia, deep vein thrombosis, pulmonary embolism, and mortality during hospitalization which is important in practice but has not been demonstrated in large database studies. For the prevention of pneumonia, the benefits of respiratory rehabilitation and oral care have been reported [20, 21]; for the prevention of deep vein thrombosis and pulmonary embolism, anticoagulation, including low molecular weight heparin, is important [22–24]. There are also systematic reviews where the benefit of pharmacotherapy is more useful than other physical measures [23]. In this study, 80% of patients in the group who had surgery on the day of admission or the next day and more than 65% of patients in the group who had surgery on the third day of admission or later started rehabilitation within three days of admission. In contrast, a detailed examination of the contents of the rehabilitation program was not possible. This study did not examine the usefulness of early rehabilitation for the prevention of pneumonia, deep vein thrombosis, or pulmonary embolism, or the mortality during hospitalization. Large-scale studies on the usefulness of early rehabilitation in preventing subsequent events are expected in the future. Anticoagulation is an independent factor and is expected to have a significant impact on the prevention of deep vein thrombosis and pulmonary embolism. In the present study, the use of anticoagulants was higher in the group that underwent surgery on or after the third day of admission. It was possible that anticoagulation may have been omitted in patients who were able to undergo surgery earlier. It was also speculated that anticoagulation for complications may be associated with delayed surgery. On the other hand, we considered the surgery results on the day of admission and the following day noteworthy, as they showed a preventive effect on pneumonia, deep vein thrombosis, pulmonary embolism, and mortality during hospitalization.

This large study has several limitations, which are described below. The study population included hip fracture patients treated only in acute care hospitals reporting to the DPC data system. This does not include patients admitted to non-DPC-reporting beds, which account for 30% of all general hospital beds, or patients who have never been treated in an acute care hospital [13]. A limitation of the study is that the time between injury and surgery has not been investigated. Although the surgical procedure should be considered within 48 h of the injury, this study considered the surgical procedure on the day of admission and the following day. Other limitations of this study were the inability to examine the severity of comorbidities, the time from surgery to the onset of sequelae and their severity, and the details of rehabilitation. Further large-scale studies based on actual patient data are needed. To examine pneumonia, deep vein thrombosis, and pulmonary embolization in patients with hip fractures in more detail, the denominator of all elderly patients with hip fractures in the community needs to be examined, and details of treatment in acute care hospitals need to be examined.

In conclusion, surgery within 48 h of injury for elderly hip fracture patients in Europe and the US has been reported to be important for good outcomes. A large DPC-based study in Japanese subjects also found that surgery on the day of admission and the following day was important in preventing complications such as pneumonia, deep vein thrombosis, and pulmonary embolism, and mortality during hospitalization. Orthopedic surgeons, as well as physicians who treat the elderly, should be aware of the importance of early surgery after hospitalization for hip fractures in the elderly.

## Abbreviations

CI: Confidence interval
DPC: Diagnosis procedure combination
PS: Propensity score
